# Transcriptome analysis reveals that Müllerian inhibiting substance regulates signaling pathways that contribute to endometrial carcinogenesis

**DOI:** 10.3892/ijo.2015.2920

**Published:** 2015-03-06

**Authors:** Youn Jee Chung, Hyun Jung Kim, Sang Ho Park, Joo Hee Yoon, Mee Ran Kim, Suk Woo Nam, David T. MacLaughlin, Patricia K. Donahoe, Jang Heub Kim

**Affiliations:** 1 Department of Obstetrics and Gynecology, College of Medicine, The Catholic University of Korea, Seoul 137-701, Republic of Korea; 2 Department of Pathology, College of Medicine, The Catholic University of Korea, Seoul 137-701, Republic of Korea; 3 Pediatric Surgical Research Laboratories, Massachusetts General Hospital, Harvard Medical School, Boston, MA 02114, USA

**Keywords:** Müllerian inhibiting substance, AN3CA cell line, transcriptome analysis, Wnt signaling pathway, cell cycle, apoptosis

## Abstract

Müllerian inhibiting substance (MIS) has been shown to inhibit growth of a number of tumors *in vitro* and/or *in vivo*, but the downstream pathways which it regulates are not fully understood. In the present study we show that MIS type II receptor was highly expressed in AN3CA cells, a cell line derived from human endometrial cancer cell in which MIS-treatment caused a reduction of cell viability, and induced cellular apoptosis and genes involved cell cycle arrest. To understand the genome-wide effects of MIS on gene regulation, we performed serial gene expression analyses from 0 to 96 h at 24 h intervals after treating AN3CA cells with MIS. Transcriptomic analysis of molecular changes induced by MIS identified 2,688 differentially expressed genes that were significantly up- or down-regulated during the 96 h study period. When the 2,688 differentially expressed genes were mapped to known biological processes, Wnt-, cancer-, proteolysis-, cytoskeleton-, cell cycle-, apoptosis-, and MAPK-signaling pathways emerged as the functions most significantly changed by MIS in AN3CA cells. Furthermore, western blot analysis validated that protein expression of cell cycle inhibitory genes, apoptotic protease activating factor-1 (APAF-1), β-catenin-interacting protein (ICAT), Rb related protein 130 (p130), and inhibitor of disheveled Dvl and Axin complex (IDAX), were gradually increased over the time of the study, whereas downstream cell cycle activating genes, cyclin-dependent kinase 2 (CDK2) and phospho-c-Jun were downregulated in MIS-treated AN3CA cells. These transcriptome analyses support previous observations that MIS functions as a tumor suppressor, potentially by regulating signaling pathways that could contribute to endometrial carcinogenesis, and indicating that MIS should be considered as a potential treatment for endometrial cancer.

## Introduction

Endometrial cancer is the most common invasive gynecologic cancer in the Western world and the fourth most common cancer in women after breast, lung and colorectal cancers (1). Endometrial cancer has been divided into two different types on the basis of distinct histology and clinical outcomes. Most endometrial cancers are type I (approximately 75%) estrogen-dependent adenocarcinomas with endometrioid morphology. Type I endometrial cancers are low-grade, early stage cancers that usually develop in perimenopausal women and coexist with, or are preceded by, complex and atypical endometrial hyperplasia, and have an excellent prognosis (2). In contrast, type II endometrial cancers are of poorly differentiated endometrioid and serous histologies that occur mainly at an older age and are associated with a lower 5-year survival rate (3). The definite role of estrogen in the development of most endometrial cancers is established; any factor that increases exposure to unopposed estrogen increases the risk for endometrial cancer. Endometrial cancers are characterized by various genetic alterations. Type I endometrial cancer harbors altered PI3K/AKT/mTOR signaling pathway constituents (4). Type II endometrial cancer have loss of function mutations in p53 (5) and gain of function mutations in epidermal growth factor receptor 2 (HER-2) (6).

Most gynecologic tumors originate from Müllerian ducts, which develop into the Fallopian tubes, uterus, cervix, proximal vagina, and from the surface epithelium of the ovary. Müllerian inhibiting substance (MIS), also known as anti-Müllerian hormone (AMH), has long been known for causing the regression of the embryonic Müllerian duct. MIS has been shown to inhibit tumor growth *in vitro* and *in vivo*, but its downstream regulated genes have not been fully elucidated. MIS initiates its effect by binding to the MIS type I and type II receptors (7). Several studies showed that MIS exerts antiproliferative and apoptotic effects on gynecologic malignancies such as cervical and ovarian cancers (8,9). In ovarian cancer, the MIS caused a suppressive effect on Cyclin Dependent Kinases (CDKs) and the E2F transcription/dimerization partner (E2F/DP1) complex, with transcriptional enhancement of certain histone deacetylases, which act as co-suppressors of the E2F/DP complex (9). In cervical cancer, MIS suppresses cell division by inducing an increase in the expression of CDK inhibitors such as p16^INK4a^ which results in a decrease in activity of the CDK complex leading to inhibition of E2F activity (8). Since endometrial cancer is also a tumor of Müllerian duct origin and prior studies (10) have reported antitumor properties of MIS against endometrial carcinoma cell lines, we sought to understand the molecular drivers of growth inhibition or apoptosis in endometrial cancer. To do so, we undertook transcriptome scans of AN3CA cells treated with MIS. Analysis of the differentially expressed genes that were further validated by western analysis indicated that MIS could elicit its tumor suppressive effects on endometrial cancer cells through control of components of pathways on which endometrial cancer progression is dependent.

## Materials and methods

### Endometrial cancer cell culture

The human endometrial cancer cell line, AN3CA (American Type Culture Collection, Manassas, VA, USA) was grown in Eagle’s minimal essential medium (Gibco, Grand Island, NY, USA) containing 10% Fetal Bovine Serum (FBS), 100 U/ml penicillin and 100 U/ml streptomycin, and incubated at 37°C in humid air with 5% CO_2_. All experiments were done on low passage cells that were cycled into a proliferative phase and harvested at 80% confluency.

### Immunohistochemical analysis of MIS Type II receptor

Immunohistochemical analysis to detect MIS RII expression was performed using the Invitrogen Histostain Plus AEC kit (Invitrogen, Carlsbad, CA, USA) according to the manufacturer’s instructions. Cultured AN3CA cells were harvested and 200 μl of a 1×10^5^ cell/ml suspension was centrifuged with Cytospin (Thermo Electron Corp., Cheshire, UK) at 1000 rpm for 5 min and attached to Probe-on-plus-slides. These slides then were treated with 3% H_2_O_2_ for 5 min to eliminate endogenous peroxidase activity and washed three times with Tris-buffered saline with 0.1% Tween-20 (T-TBS). After treatment with donkey serum (Invitrogen) for 30 min to block non-specific protein binding, the slides were incubated with rabbit polyclonal anti-human MIS type II receptor antiserum (Massachusetts General Hospital, Boston, MA, USA) (11) at 4°C overnight. The slides were rinsed in T-TBS three times, and incubated with biotinylated anti-rabbit IgG (Invitrogen) for 30 min. After another three T-TBS rinses, the streptavidin HRP detection system (Invitrogen) was applied to the slides for 30 min to induce the biotin-avidin binding reaction. The slides were treated with 3-amino-9-ethylcarbazole (AEC, Invitrogen) for 10 min at room temperature, counterstained with hematoxylin, then mounted with glycerol gel.

### Methylthiazoletetrazolium (MTT) assay for cell viability

Three thousand cells/well low passage cells that were subcultured three times then harvested at 80% confluency were seeded in 96-well plates in Dulbecco’s modified Eagle’s medium (DMEM) with 10% FBS. After 24 h the cells were exposed to 71.4 nM MIS for 0, 24, 48, 72 or 96 h, or the equivalent volume of phosphate buffered saline (PBS, pH 7.4) as a vehicle control. The treated cells were washed with PBS and 100 μl of MTT solution [5 mg/ml MTT stock (Roche Molecular Biochemicals, Mannheim, Germany) in PBS diluted to 1 mg/ml with 10% DMEM] was added to each well. Cells were incubated for 4 h at 37°C at the end of which 200 μl DMSO was added for 30 min at room temperature in the dark. Optical densities at 550 nm were measured using an ELISA plate reader (Bio-Tek Instruments, Winooski, VT, USA).

### Cell cycle analysis

AN3CA cells managed as above were exposed to 71.4 nM MIS (Massachusetts General Hospital) (12) for 0, 24, 48 or 72 h, or the equivalent volume of PBS (pH 7.4) as a vehicle control and the treated cells were collected by trypsinization. The cells were fixed with 100% methanol and stored for 30 min at 20°C. The cells were washed with PBS, centrifuged, resuspended in 1 ml DNA staining solution (20 μg/ml propidium iodide, 200 μg/ml DNase free RNase), incubated in the dark at 37°C for 30 min, and then analyzed on a FACSVatage SE Flow Cytometer (Becton Dikison, San Jose, CA, USA). The forward scatter and red fluorescence >600 nm were measured and the results analyzed using CellQuest software and Modfit LT 3.0 program (Verity Software House, Topsham, ME, USA).

### Annexin V analysis

AN3CA cells managed as above were treated with 71.4 nM MIS for the same intervals then harvested and stained for Annexin V and propidium iodide (PI) using the Annexin V-FITC Apoptosis Detection kit I (BD Biosciences, San Diego, CA, USA) according to the manufacturer’s protocol. Brielfy, following drug treatment, 10^5^ cells were pelleted, washed once with PBS, and resuspended in 100 μl of binding buffer [10 mM HEPES (pH 7.4), 150 mM NaCl, 5 mM potassium chloride, 1 mM MgCl_2_, and 2 mM calcium chloride]. Subsequently, 5 μl of Annexin V-FITC and PI was added to the cells which were then incubated for 15 min at room temperature in the dark. After incubation, 400 μl of binding buffer was added to the stained cells which were analyzed using a FACSVatage SE flow cytometer (Becton Dickinson). Data analysis was conducted using CellQuest software.

### RNA isolation and gene profiling

Total RNA was extracted from the AN3CA cells managed as above with TRIzol reagent (Invitrogen), and the purity and yield of the RNA were determined spectrophotometrically. The quality of total RNA was analyzed by using the RNA StdSens Chips on the Experion™ system (Bio-Rad, Hercules, CA, USA). Microarray analysis was performed by using Human HT-12 v4 Expression BeadChip (Illumina, Inc., San Diego, CA, USA). The RNA was processed by using the Illumina RNA Amplification kit (Ambion, Inc., Austin, TX, USA) according to the manufacturer’s instructions starting with 1.1 μg total RNA. Resulting biotin-labeled cRNA was recovered and purified with RNeasy kit (Qiagen, Valencia, CA, USA), hybridized to the chips, and fluorescently tagged and scanned by using Illumina BeadStation (Illumina, Inc.) according to the manufacturer’s protocol. All arrays were run in the array core facility of the Functional RNomics Research Center at the Catholic University of Korea.

### Microarray data processing and analysis for gene expression

BeadStudio (version 3.0) was used for data acquisition and calculation of signal values on Illumina expression microarray. Normalization of microarray data and hierarchical clustering were performed by using GenPlex™ (version 3.0). Sets of differentially expressed genes were identified by a parametric test (Welch’s t-test). A threshold P-value in combination with fold change was applied. Expression profiles of the gene sets with a fold change regulation of more than 1.3-fold and P<0.05 were examined to find the differentially expressed genes. Hierarchical clustering was performed by using Cluster and TreeView 2.3 (Stanford University). Euclidean correlation, median centering, and complete linkage were applied during all clustering applications.

### Western blot analysis

Proteins from 10 μg/ml of MIS treated cells were harvested in radioimmunoprecipitation assay (RIPA) buffer (150 mM NaCl, 1% NP-40, 0.5% sodium deoxycholate, 0.1% SDS, 50 nM Tris-HCl) with 1 μM phenylmethylsulfonyl fluoride and protein concentration was determined by bicinchoninic acid protein assay reagent (Thermo Scientific, Waltham, MA, USA). Equal amounts of protein were separated on sodium dodecyl sulfate (8–12% gel) polyacrylamide gels (50 μg per lane), and transferred to polyvinylidene difluoride membrane. The blots were blocked in TBS-T (20 mM Tris-HCl, pH 7.6, 137 mM NaCl, 0.1% Tween-20) containing 5% powdered milk for 1 h and then incubated in 1% milk TBS-T with the primary antibodies at 4°C overnight. Apaf-1 (sc-8339, Santa Cruz Biotechnology, Santa Cruz, CA, USA), caspase-3 (9668, Cell Signaling Technology, Inc., Boston, MA, USA), CDK2 (sc-6248, Santa Cruz Biotechnology), ICAT (sc-99240, Santa Cruz Biotechnology), Idax (sc-164631, Santa Cruz Biotechnology), PARP (9542, Cell Signaling Technology, Inc.), phospho-c-Jun (3270s, Cell Signaling Technology, Inc.), p107 (sc-318, Santa Cruz Biotechnology) and p130 (sc-317, Santa Cruz Biotechnology) were used at a 1:200 dilutions. Blots were then washed three times with 1% milk TBS-T, incubated with the corresponding horseradish peroxidase-conjugated secondary antibody, and detected using the Pierce ECL western blotting substrate (Thermo Scientific).

### Statistical analysis

Results are presented as mean ± SD of replicate analyses and are either representative of, or inclusive of, at least three independent experiments. Statistical comparisons between two experimental groups were performed using Student’s t-test (paired) whilst multiple group comparisons were performed using analysis of variance (ANOVA). Data were regarded as statistically significant when P<0.05.

MTT results are presented as percentage of control, which was calculated using the following equation: (mean absorbance of treated cells/mean absorbance of control cells) ×100. Data are expressed as mean ± SD from nine independent experiments, each with replicates of three. A P-value <0.05 was considered statistically significant when compared with corresponding vehicle control cells.

Cell cycle distributions after exposure of AN3CA endometrial cancer cells to MIS are presented as histograms of the mean ± SD from three independent experiments. Annexin V analysis was done to evaluate apoptosis. Quadrant rectangular dot grams from a representative of three independent experiments were analyzed.

## Results

### Treatment results of Müllerian inhibiting substance in growth retardation and cellular apoptosis of AN3CA endometrial cancer cells

**Figure 1:**
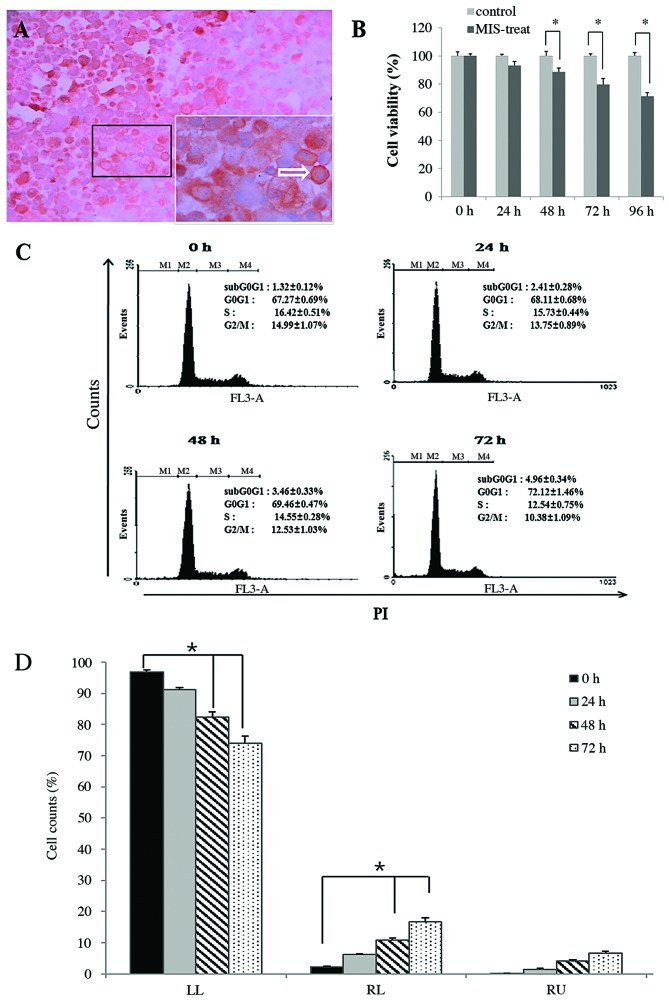
(A) Expression of MISRII on AN3CA endometrial cancer cells as assessed by immunocytochemistry using an MISRII antibody. AN3CA cells showed strong specific MISRII staining, which was the most intense at the cell surface (x200). At higher magnification (x400) the arrow in the boxed area shows strong surface expression of MISRII on AN3CA cell (characteristic of n=3). (B) Inhibition of cell growth by MIS in AN3CA cells was assessed by the cell viability MTT assay. AN3CA cells were treated with 10 μg/ml (71.4 nM) MIS for 0, 24, 48, 72 and 96 h, stained with MTT, and the absorbance was read at 550 nm. Results are presented as a percentage of the untreated control which was calculated as mean absorbance of treated cells/mean absorbance of control cells ×100 and expressed as mean ± standard deviation (SD) from three independent experiments (^*^P<0.05 as compared to corresponding control cells). (C) To determine cell cycle distribution after exposure to MIS, AN3CA endometrial cancer cells were treated with 10 μg/ml MIS for 0, 24, 48 and 72 h, trypsinized and fixed in 100% methanol. Then washed cells were exposed to propidium iodide/RNase solution and histograms of cellular DNA content obtained by flow cytometry (representative of 3 replicates). (D) To detect induction of apoptosis by MIS, AN3CA endometrial cancer cells were treated with 10 μg/ml MIS for 0, 24, 48 and 72 h, respectively, and externalization of phosphatidylserine assessed by measuring Annexin V-FITC binding using propidium iodide as a counterstain (LL, surviving cells; RL, early apoptotic cells; RU, late apoptotic and necrotic cells, ^*^P<0.05 as compared to corresponding 0 h, representative of 3 replicates).

It has been well established that proliferation of ovarian, cervical and breast cancer cells are suppressed by MIS treatment, and many of these studies mentioned the potential of MIS as a treatment of MIS-receptor expressing tumors. Therefore, before initiating experiments on the regulatory roles of MIS in endometrial cancer cells, it was necessary to confirm expression of the MIS receptor in AN3CA cells. To this end, we performed immunohistochemical staining with an anti-MIS type II receptor (MISR II) antibody on AN3CA cells which detected strong expression on their cell surface (Fig. 1A).

Next, to investigate the antitumor effects of MIS on endometrial cancer cells, AN3CA cells were treated with MIS at the indicated concentration for 0, 24, 28, 72 and 96 h, and MTT assays were performed. As shown in Fig. 1B, MIS treatment on AN3CA cells resulted in a 30% reduction of tumor cell growth (n=3, P<0.05, using Student’s t-test) resulting from cell cycle arrest or apoptosis. Cell cycle analysis of PI-stained cells using flow cytometry at 72 h after MIS treatment (Fig. 1C) detected a slight 4.85% increase in cells in the G1 phase, with a concomitant decrease in S phase and G2/M phase by 3.88 and 4.61%, respectively, compared to controls (non-treatment) (n=3, P<0.05, using Student’s t-test). Flow cytometric analysis at 72 h of Annexin V stained AN3CA cells (Fig. 1D), however, showed a statistically significant increase (n=3, P<0.0001, using Student’s t-test) in early cellular apoptosis of 14.37% (from 2.31±0.18% at 0 h to 16.68±1.21% at 72 h) and in late cellular apoptotic and necrotic cells of 6.49% (from 0.04±0.03% at 0 h to 6.53±0.75% at 72 h) (Fig. 1D). These results suggest that MIS exerts its antitumor effects on endometrial cancer cells by causing significant cellular apoptosis.

### Characterization of molecular and functional signatures after MIS treatment of endometrial cancer cells

**Figure 2:**
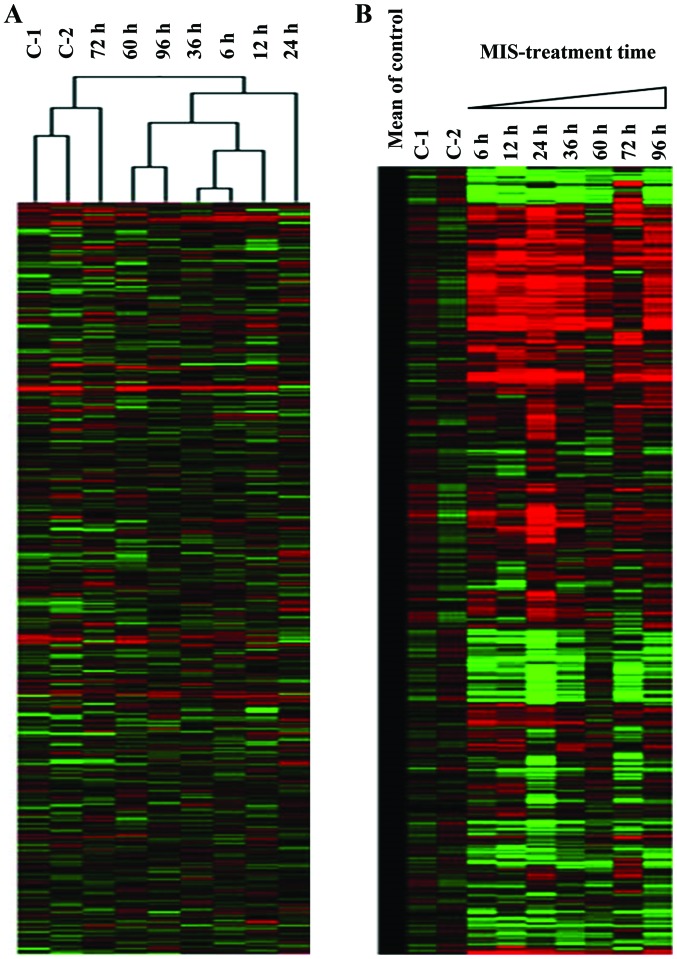
Differential gene expression profiling and identification of molecular changes in the AN3CA cells that were treated with 10 μg/ml MIS are shown by (A) unsupervised hierarchical clustering of 11,470 genetic elements with minimum selection and filtering criteria. (B) Mathematical comparisons of genes between 0 h and the 6–96 h time points. Selected 2,688 genes showing at least 1.3-fold changes induced by MIS treatment compared to non-treated control (0 h) are visualized as a heat map where red are higher, and green are lower than the mean value of non-treated cells.

To identify a molecular signature induced by MIS, serial gene expression analysis was conducted. AN3CA cells were continuously treated with MIS for up to 96 h, and the RNAs were harvested for microarray analysis at multiple time points from 6 h. In Fig. 2A, 11,470 genetic elements, which passed the minimum selection and filtering criteria, are shown in unsupervised hierarchical clustering. Mathematical comparisons of genes between 0 h and the 6–96 h time points were performed and 2,688 genes showing at least 1.3-fold changes (Fig. 2B) were visualized as a heat map where red indicates that expression levels of genetic elements that are higher after MIS treatment and green indicates that expression levels of genetic elements that are lower than the mean value of no treatment.

**Figure 3:**
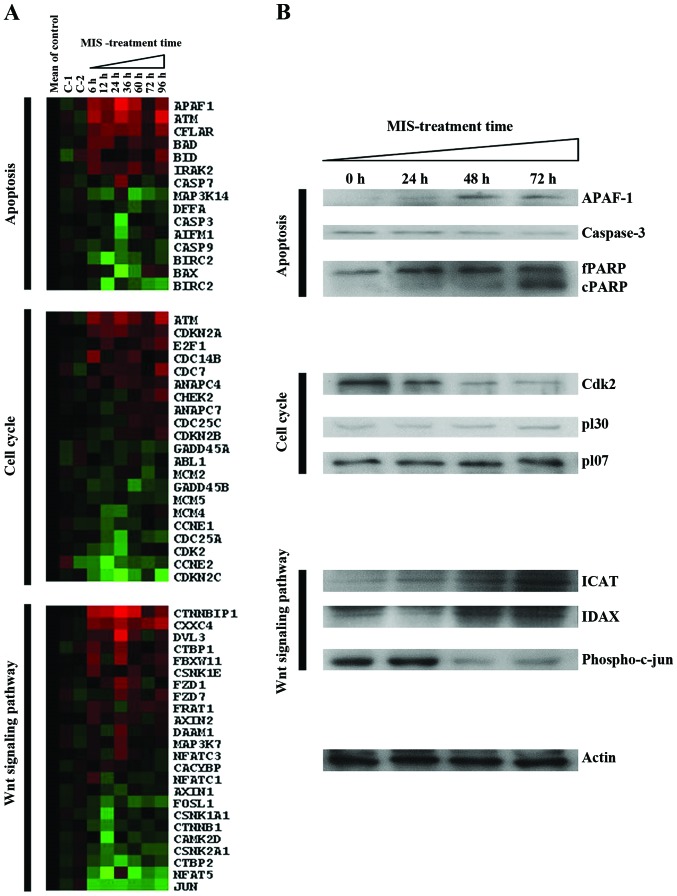
(A) Selective expression profile of KEGG pathway-related genetic elements retrieved from 2,688 differentially expressed genes. Fifteen genes were mapped to apoptosis, 21 to cell cycle and 24 to Wnt signaling pathways in the KEGG database. These elements were then visualized as a heat map of expression of genetic elements where red are higher, and green are lower than the mean value of non-treated cells. (B) Western blot analysis for the expression of apoptosis, cell cycle and Wnt signaling pathway-related proteins in AN3CA cells treated with 10 μg/ml MIS for 0, 24, 48 and 72 h, representative of 3 replicates.

To derive insight into the molecular pathways in which these 2,668 genes participate, we used the pathway tool in the Kyoto Encyclopedia of Genes and the Genomes (KEGG) pathway database (http://www.genome.jp/kegg/) which maps genes to known pathways and provides a summary of the biological processes affected. Based on this database analysis, we identified 20 pathways containing at least 10 genetic elements from the 2,668 differentially expressed genes. AN3CA cells treated with MIS fall into cancer-related signaling pathways involving Wnt, cell cycle, MAPK, p53 and apoptosis pathways (Table I). From these, genes associated with Wnt, cell cycle regulation and apoptosis pathways were depicted as heat maps (Fig. 3A). In Wnt signaling pathways, CTNNBIP1 and CXXC4 genes were upregulated and JUN was downregulated compared to non-treatment. CDK2 gene was downregulated in cell cycle pathways. In apoptosis pathways, the APAF1 gene was upregulated while caspase-3 was slightly downregulated (Fig. 3A).

Differentially expressed genes in AN3CA cells, that were examined by western analysis include phospho-c-Jun, ICAT, IDAX, CDK2, p130, p107, APAF-1, caspase-3 and PARP, which confirmed upregulation of ICAT and IDAX and downregulation of phospho-c-Jun in Wnt signaling pathways, downregulation of CDK2 and slight upregulation of p130 and p107 in cell cycle pathways, and upregulation of APAF-1 and cPARP and downregulation of full length caspase-3 in the apoptosis pathway (Fig. 3B).

## Discussion

In previous studies, MIS has been shown to inhibit the growth of ovarian, cervical and endometrial cancer cell lines. Treatment with recombinant human MIS induced upregulation of both p107 and p130 in non-HPV-related cervical cancer cell lines (13) and inhibited the growth of the ovarian cancer cell line through induction of the CDK inhibitor p16 at the protein level and downregulation of p130 and upregulation of E2F1 when Rb levels were not detectable (14). In endometrial cancer cell lines (AN3CA) lacking p16 and pRb, MIS-induced apoptosis was associated with an increase in p107 and p130 and a decrease in E2F1 expression at 72 h (10). Thus, gene regulation by MIS appears to be specific to tumor type, but in these studies involves regulation of cell cycle pathways.

The cornerstone of curative therapy for endometrial cancer is surgical treatment, including complete hysterectomy, adnexectomy and appropriate surgical staging in patients considered at high risk. The type of surgery and postoperative therapy depends on the stage and other clinicopathological risk factors and survival is strongly dependent on surgical stage. Recent advances in the understanding of the molecular and genetic basis of endometrial cancer have led to the development of targeted therapies that inhibit angiogenesis and the cellular signaling pathways involved in cell growth and proliferation. Trastuzumab, a human EGFR type II (HER2)-related inhibitor that affects signal transduction, is currently a standard treatment for HER2-positive breast cancer. Certain serous adenocarcinomatous endometrial cancers which are characterized by overexpression of HER2 could be helped by trastuzumab (15). mTOR inhibition with temsirolimus has encouraging single-agent activity in endometrial cancer which has been shown to be more effective in chemotherapy-naive patients than in chemotherapy-treated patients and is independent of *PTEN* status (16). Another mTOR inhibitor, ridaforolimus, has antitumor activity and acceptable tolerability in advanced endometrial cancer patients (17). Given the potential of MIS as a therapeutic for endometrial cancer, particularly the more aggressive Type II, it is important to understand the genes regulated by MIS.

To demonstrate the molecular mechanism by which MIS might inhibit the growth of an endometrial carcinoma cell line, we first evaluated the expression of MISRII on the cell surface of the endometrial carcinoma cell line, AN3CA. After confirmation of the MISRII expression on the AN3CA by immunohistochemistry, we examined the inhibitory effect of MIS on the growth of the endometrial cell line and showed 7–29% reduction in cell survival, an increase of cellular apoptosis and a small change in cell cycle parameters. Microarray and western blot analyses confirmed changes in pathways related to cell cycle, apoptosis and Wnt signaling.

For example, ICAT, the protein encoded by CTNNBIP1, a negative regulator of the Wnt signaling pathway (18) binds CTNNB1 (β-catenin) and prevents its intracellular interaction with TCF (T-cell transcription factor) family members. IDAX, the protein encoded by the CXXC4 gene also functions as a negative regulator of the Wnt signaling pathway by directly binding to the PDZ domain of Disheveled (DVL) in the nucleus (19). C-Jun transcribes a protein that is required for progression through the G1 phase of the cell cycle as c-Jun null cells show increased G1 arrest (20). C-Jun is also a target gene of β-catenin, which, if decreased, could result in tumor suppression, indicating that MIS could possibly result in tumor suppression, through regulation of Wnt signaling.

APAF1 encodes a cytoplasmic protein that forms one of the central hubs in the apoptosis regulatory network, and its activation of PARP induces necrotic and programmed cell death, thus supporting the most robust changes seen in these studies (Fig. 1D, i.e., apoptosis). In previous studies, MIS has been documented to increase both p107 and p130 in AN3CA cells (10), which was also correlated with inhibition of proliferation in cervical cancer cell lines (13). Slight upregulation of p107 and p130 was shown in the study. However, other genes associated with cell cycle demonstrated significant differences after MIS treatment; in cell cycle pathways, CDK2 is essential for the G1/S transition and reduction of CDK2 levels correlates with cell cycle arrest.

In summary, these findings indicate that MIS regulates the proliferation of tumor cells through modulation of apoptosis and cell cycle pathways, and by causing blockade of the Wnt/β-catenin signaling pathways. The findings support the hypothesis that MIS was able to function as a potential therapeutic for endometrial cancer by targeting these molecular pathways.

## Figures and Tables

**Table I:** Summary of the KEGG pathways that are part of the characteristic molecular signature of MIS in AN3CA cells.

KEGG pathway	Gene counts	Upregulated genes	Downregulated genes
Pathways in cancer	41	17	24
MAPK signaling pathway	35	17	18
Alzheimer’s disease	23	12	11
Regulation of actin cytoskeleton	23	7	16
Ubiquitin mediated proteolysis	21	14	7
Focal adhesion	21	4	17
Wnt signaling pathway	20	8	12
T cell receptor signaling pathway	17	6	11
GnRH signaling pathway	16	8	8
Chronic myeloid leukemia	15	4	11
Axon guidance	14	6	8
Colorectal cancer	14	4	10
Huntington’s disease	14	5	9
ErbB signaling pathway	14	4	10
Prostate cancer	14	4	10
Apoptosis	14	7	7
Pathogenic *Escherichia coli* infection	14	1	13
Cell cycle	13	4	9
p53 signaling pathway	11	4	7
Tight junction	11	4	7

There were significant pathways including more than 10 genes by one pathway in outliers of 2,688 genes which showed expression more than 1.3-fold increase or decrease.
